# Effects of Tai Chi on insomnia in elderly people with chronic non-specific low back pain: A study protocol for a randomized controlled trial

**DOI:** 10.3389/fpsyg.2023.1105359

**Published:** 2023-02-24

**Authors:** Feng Zhang, Boran Zhang, Xiaotong Wang, Chang Huang, Boyi Hu

**Affiliations:** ^1^School of Acupuncture-Moxibustion and Tuina, Beijing University of Chinese Medicine, Beijing, China; ^2^Department of Physical Education, Beijing University of Chinese Medicine, Beijing, China

**Keywords:** Tai Chi, insomnia, elderly people, randomized controlled trial, chronic non-specific low back pain

## Abstract

**Introduction:**

Chronic non-specific low back pain (CNLBP) is a complex condition characterized by pain, dysfunction, disturbed sleep, anxiety, and depression, all of which impair the quality of life. Previous studies showed that practicing Tai Chi had effects on chronic low back pain. However, there is a lack of evidence on its impact on sleep. The trial will evaluate the use of Tai Chi as a treatment for insomnia in elderly people with CNLBP.

**Methods:**

The study design will be a randomized, controlled, open-label trial. Participants (*n* = 106) will be recruited from the Hospital of Chengdu University of Traditional Chinese Medicine, Qing Yang District University for the Elderly, and Ci Tang Street Community. Participants will be randomly assigned to the Tai Chi group (*n* = 53) and the control group (*n* = 53). The Tai Chi group will undergo a Yang-style 24-form Tai Chi program for 8 weeks. The control group will have a waiting period of 8 weeks, followed by 8 weeks of Tai Chi practice. The primary outcomes of this study will be changes in sleep quality and pain intensity. Secondary outcomes of interest will include changes in the quality of pain, range of motion, physical performance, social support, and overall quality of life. Any adverse events and attendance rates will also be reported in this study.

**Clinical trial registration:**

ChiCTR2200064977.

## Introduction

Low back pain is a public health problem that primarily affects women between the ages of 40 and 80 worldwide (Hill et al., 2011; Hoy et al., 2012; Knezevic et al., 2021). The prevalence of low back pain is 84%, and the disability rate is 11–12% (Albazli et al., 2021). Low back pain is a significant source of social and economic burden (Comachio et al., 2015). The disease's global burden is expected to rise due to an increase in the aging population and population growth (DALYs and Collaborators, 2016). Low back pain presents in two forms: non-specific or mechanical (Will et al., 2018). Chronic non-specific low back pain (CNLBP) is defined as persistent pain that lasts for more than 12 weeks and that occurs in the lower part of the spine between the dorso-lumbar and the lumbosacral hinges. It is not associated with specific pathologies, such as infections, tumors, osteoporosis, or lumbar spine fractures (Balague et al., 2012; Mu et al., 2020; Kabore et al., 2022). CNLBP affects up to 90% of patients, with a prevalence of 2–7% among those who suffer from chronic pain (Bellido-Fernandez et al., 2022). CNLBP is the result of a complex interplay between physical factors (such as disability, muscular strength and endurance, and body composition), psychological factors (such as pain self-efficacy, depression, anxiety, and sleep quality), and social factors (such as social functioning and support networks) (Iglesias-Gonzalez et al., 2013; Rodrigues-De-Souza et al., 2016; Lall and Restrepo, 2017; Volpato et al., 2019; Tagliaferri et al., 2020; Ho et al., 2022). It is the greatest cause of disability and is associated with age, sleep quality, BMI, smoking habits, and co-morbidity (Sidiq et al., 2021). Insomnia and disrupted sleep negatively affect pain, functional ability, anxiety, depression, and the quality of life of patients with CLBP (Akodu and Akindutire, 2018; van de Water et al., 2011). According to a study by Marin et al. (2006), individuals with CNLBP experienced a 55% decrease in sleep quality when their pain increased. Evidence indicates that participants with CLBP are at 2.4 times greater risk of experiencing sleep problems (Stubbs et al., 2016). Poor sleep quality in participants with CLBP is moderately correlated with physical disability (McCracken and Iverson, 2002). Therefore, improving sleep quality may be beneficial for patients with CNLBP. A study by Bedaiwi et al. (2016) showed that, while celecoxib had a protective effect on patients with CNLBP, this treatment did not result in any changes in the inflammatory lesions of the sacroiliac joints or the spine. A prospective study has indicated that non-steroidal anti-inflammatory drugs (NSAIDs) have therapeutic effects on patients with CNLBP (Takahashi et al., 2017). However, 3–23% of patients discontinue NSAID therapies owing to their side effects (Schnitzer et al., 2004). Chung et al. (2013) found that opioids and partial opioid agonist drugs provide pain relief in patients with CNLBP. Several studies have reported the use of opioids to treat CNLBP. However, they are often accompanied by insomnia (Benca et al., 1992; Benca, 1996; Latif et al., 2019). Evidently, pharmacotherapy alone is insufficient to effectively treat insomnia in patients with CNLBP. Clinical guidelines indicate that self-management, exercise therapy, and cognitive behavioral therapy have been shown to have positive effects on CLBP (Savigny et al., 2009; Koes et al., 2010; Eadie et al., 2013). A recent randomized controlled trial found that moderate-intensity exercise training has a positive effect on sleep quality (Tseng et al., 2020). However, research examining the effectiveness of exercise therapy in improving insomnia in patients with CNLBP is scarce.

Tai Chi, developed a few hundred years ago, is a traditional Chinese mind-body practice that combines physical, psychosocial, and social factors to promote health. It is an ancient discipline characterized by flowing circular movements of the upper limbs and constant weight shifting in the lower limbs (Peng, 2012; Dong et al., 2022; Song et al., 2022). Tai Chi has five forms: Chen, Yang, Wu, Sun, and Wu/Hao. Tai Chi is an exercise of mild-to-moderate intensity for older people (Siu et al., 2021a). Several studies have investigated the effects of Tai Chi on Parkinson's disease, diabetes, anxiety, depression, and so on Yeh et al. (2009); Li et al. (2012); Liu et al. (2020). Recent studies report Tai Chi to be a viable alternative for managing insomnia (Siu et al., 2021b). Tai Chi has been shown to have beneficial effects on insomnia in breast cancer survivors (Irwin et al., 2017). The study concluded that Tai Chi can effectively improve pain and disability in people with persistent low back pain (Hall et al., 2011; Liu et al., 2019). Tai Chi also has protective effects on the neuromuscular function of the lower extremities in aging patients with CNLBP (Zou et al., 2019). Tai Chi may be a potential alternative method to affect cognitive appraisal outcomes (Hall et al., 2016). However, it is unknown whether Tai Chi is an effective treatment for insomnia in patients with CNLBP.

We will conduct a randomized controlled trial to evaluate Tai Chi's effectiveness in managing insomnia in elderly people with CNLBP. We hypothesize that Tai Chi will be more effective in improving insomnia, pain, and physical and social functioning than the control group.

## Methods

### Study design

This study design conducts an open-label, randomized, controlled trial. The study protocol was approved by the Ethics Committee of the Hospital of Chengdu University of Traditional Chinese Medicine in Sichuan Province on 06/06/2022 (ethics approval number: 2022KL-038-02). This trial is registered with the Chinese Trial Registry under the number ChiCTR2200064977. This protocol adheres to the Consolidated Standard of Reporting Trials (CONSORT) and the Standard Protocol Items: Recommendations for Interventional Trials (SPIRIT). This study will be conducted at the Hospital of Chengdu University of Traditional Chinese Medicine, Qing Yang District University for the elderly, and Ci Tang Street Community. We will compare the effects of the Tai Chi group against the control group on insomnia in elderly people with CNLBP. Baseline assessments and follow-up assessments will be conducted at weeks 8, 16, 32, and 40 to measure primary outcomes such as sleep and pain intensity as well as secondary outcomes such as quality of pain, range of motion, physical performance, social support, quality of life, attendance rate, and adverse events. The detailed schedule of patient interventions and outcomes is described in Table 1.

**Table 1:** Schedule of patients, intervention, outcomes.

**Item**	**Week 0**	**Week 1–8**	**Week 8**	**Week 9–16**	**Week 16**	**Week 32**	**Week 40**
**Patients**							
Informed consent	×						
Inclusion criteria	×						
Exclusion criteria	×						
**Intervention**							
Tai Chi group		×					
Control group		×		×			
**Outcomes**							
PSQI	×		×		×	×	×
VAS	×	×	×	×	×	×	×
Quality of pain	×		×		×	×	×
Range of motion	×		×		×	×	×
JOA	×		×		×	×	×
ODI	×		×		×	×	×
CPSS	×		×		×	×	×
SSRS	×		×		×	×	×
SF-36	×		×		×	×	×
Adverse events			×		×	×	×
Attendance rate			×		×	×	×

PSQI, Pittsburgh Sleep Quality Index; VAS, Visual Analog Score; JOA, Japanese Orthopedic Association Scores; ODI, Oswestry Disability Index; CPSS, Chronic Pain Self-Efficacy Scale; SF-36, Short Form 36; SSRS, Social Support Rating Scale.

### Participants

#### Sample size

This study will be designed to compare the effectiveness of the Tai Chi group against the control group. Sample size calculations will be based on the change in the Pittsburgh Sleep Quality Index (PSQI) and the Visual Analog Scale (VAS) scores. The study will detect a 5-point difference between groups on the PSQI scores (Irwin et al., 2008) and a 1.96-point difference in the VAS scores (Bower and Irwin, 2016). A total sample size of 96 participants will achieve 90% power with an alpha of 0.05. However, considering a 10% drop rate, we plan to recruit 106 patients.

#### Inclusion criteria

To be included in the study, the participants must meet the following criteria:

(a) be between 60 and 75 years of age;
(b) diagnosed as having CNLBP as defined by the American College of Physicians and the American Pain Society. CNLBP is characterized by pain in the waist and the lumbosacral region, mainly located below the rib margin of the back and above the hip fissure, and may be accompanied by thigh pain, lumbar weakness, lumbar stiffness, limited lumbar mobility, with no nerve root compression, spinal canal stenosis, and trauma (Chou et al., 2007; Chen et al., 2022).
(c) suffering from CNLBP for more than 3 months;
(d) having no infectious inflammation, fractures, or tumors in the lower back; and
(e) willing to sign informed consent.

#### Exclusion criteria

Participants will be excluded from the study if they:

(a) have severe joint pain or deformity or severe spinal deformity;
(b) have serious diseases that affect mobility, such as myocardial infarction or stroke, congestive heart failure, severe chronic obstructive pulmonary disease, cancer, and other serious diseases; and
(c) have had any Tai Chi training or have participated in other clinical trials.

#### Recruitment

Advertisement posters displaying the inclusion criteria will be used to recruit participants *via* WeChat. Additionally, we will conduct educational lectures about Tai Chi at the Qing Yang District University for the Elderly and Ci Tang Street Community to raise awareness and attract qualified participants. Eligible participants will provide written informed consent.

### Procedures

#### Randomization and blinding

Eligible participants will be randomly assigned to two groups in a 1:1 ratio. Randomization will be performed by an independent researcher and will be generated in blocks of varying sizes and stratified according to the central computer system. The group assignments will be hidden in opaque envelopes by an independent researcher who will not participate in the study. The envelopes will be opened for each participant after the completion of the assessments. The instructors and participants will not be blinded to the group assignments. Research assessors and statisticians will be blinded to group allocation.

### Interventions

#### Tai Chi group

Participants in the Tai Chi group will attend a Yang-style 24-form Tai Chi training program. Each Tai Chi session will last 60 min (10 min warm-up, a 40 min Tai Chi training program, and a 10 min cooldown) three times a week for 8 weeks. The training sessions will be conducted by three experienced instructors.

#### Control group

The participants in the control group will not receive any interventions for 8 weeks. After 8 weeks, participants will be instructed to do Tai Chi exercises for 8 weeks for 60 min (10 min warm-up, a 40 min Tai Chi training program, and a 10 min cooldown) three times a week. Tai Chi group coaches will also instruct participants.

### Outcome measurements

#### Primary outcomes

##### Sleep

The primary outcome is the PSQI (Pittsburgh Sleep Quality Index) scores, which are the most commonly used measure of sleep quality and consist of 19 questions about sleep habits over a 1-month period (Nadal-Nicolas et al., 2020). The PSQI measures sleep quality, sleep latency, sleep duration, habitual efficiency, disturbances, use of sleeping medications, and daytime dysfunction (Pachikian et al., 2021). The scores range from 0 to 21, with higher scores indicating worse sleep quality. A score >5 indicates clinical sleep impairment (Kalmbach et al., 2020).

##### Pain intensity

The VAS (Visual Analog Score) is a commonly used tool for evaluating back pain. The scale ranges from 0 to 10 (Shu and Zhang, 2022), with a higher score indicating greater pain intensity. Participants will be asked to rate their pain weekly before follow-up, and the levels of pain intensity will be categorized as none (0), mild (1–3), moderate (4–6), and severe (7–10) (Liu et al., 2022).

#### Secondary outcomes

##### Quality of pain

The quality of pain will be assessed using a pain diary, which consists of the frequency and duration of pain. Patients will be instructed to maintain a daily log of the frequency of their pain and the number of times they experience it. We will monitor participants' use of the telephone to write pain diaries.

##### Range of motion

The range of motion includes lumbar flexion, extension, and right and left lateral flexion; lumbar spine rotation will be measured using a goniometer (Dayanir et al., 2020; Ricci et al., 2022). The participants will be asked to bend forward and backward into full lumbar flexion. We will measure the lateral flexion from the finger to the floor at the maximal degree (Elshiwi et al., 2019). The participants will be instructed not to move the waist and to minimize rotation as much as possible.

##### Physical performance

Physical performance will be measured using the Japanese Orthopedic Association Score (JOA, range 0–29, with higher scores indicating a greater function), the Oswestry Disability Index (ODI, range 0–100, with higher scores indicating a worse impact on life), and the Chronic Pain Self-Efficacy Scale (CPSS, range 0–110, with higher scores indicating a greater confidence in performing physical activities). The JOA score has been widely used to assess function after intervention for low back pain (Ma et al., 2021). It consists of subjective symptoms, clinical signs, daily activities, and bladder function (Tsukayama et al., 2002). The questionnaire includes 10 categories: waist and leg pain, personal life care, lifting heavy objects, walking, standing, sitting, sleeping, sexual life, social life, and travel (Marti-Salvador et al., 2018). The CPSS will be used to assess the participants' perceived self-efficacy in coping with chronic pain (Anderson et al., 1995). This scale contains self-efficiency for pain management (PSE), self-efficiency for physical function (FSE), and self-efficiency for symptoms and coping with symptoms (CSE).

##### Social support

Social support will be assessed using the Social Support Rating Scale (SSRS). Social support evaluations include objective support, subjective support, and utilization of social support. Higher scores indicate greater social support.

##### Quality of life

The participants' health status will be assessed with Short Form 36 (SF-36), which ranges from 0 to 100, with higher values indicating better general health status (Mahdavi et al., 2022). The questionnaire contains sections on physical functioning (PF), role-physical (RP), bodily pain (BP), general health (GH), vitality (VT), social functioning (SF), role-emotional (RE), mental health (MH), and health transition (HT) (Huang et al., 2019).

##### Monitoring of adverse events

The participants will be asked about the occurrence of adverse events during the observation period. We define adverse events as increased pain, falling, disability, life-threatening events, and any events related to Tai Chi. If sleep and pain continue to worsen, a medical diagnosis shall be made as soon as possible, and the clinical trial may be terminated.

##### Attendance rate

The attendance rate of Tai Chi training will be monitored by instructors. Sign-in sheets will be used to monitor attendance.

##### Data collection

The reviewer will record data using the Research Electronic Data Capture (REDCap), which is a password-protected system. All data will be double-checked by a second reviewer. Participants will be contacted about the study outcomes in the case of missing data.

### Statistical analysis

The statisticians and researchers will establish a statistical plan. It will contain baseline characteristics, treatment effects, adverse events, and attendance evaluations. All analyses will be conducted using the intention-to-treat method. Two independent sample t-tests will be used for analysis. Repeated measures analysis of variance will be used to examine the effects of VAS between the Tai Chi group and the control group. We will use the Wilcoxon-Signed Rank Test to assess non-normally distributed data. Continuous data will be expressed as mean ± standard deviation. The chi-square test will be used for categorical data. We will set the statistical significance at a *P*-value of < 0.05. Missing data will be processed according to the last observation carry-forward rules. We will use IBM SPSS Statistics version 25 (SPSS, Inc., USA) for data analysis.

## Discussion

This study will compare the effectiveness of Tai Chi in improving insomnia symptoms in elderly people with CNLBP in the Tai Chi group with that in the control group. Previous research showed that Tai Chi provides various benefits for older adults with CLBP, including improvements in functional abilities (such as balance, leg strength, and flexibility), psycho-spiritual support, and social support (Lee et al., 2020). That study was conducted with 18 participants, who were randomized into the Tai Chi group. In our study, 122 participants will be recruited. Previous studies suggest that Tai Chi has protective effects on neuromuscular function in aging individuals with non-specific low back pain, but no follow-up was reported (Zou et al., 2019; Liu et al., 2020). Our study will fill this gap by conducting a 24-week follow-up to evaluate the effects of Tai Chi on insomnia in people with CNLBP. Previous studies mostly focused on the functional benefits of Tai Chi (Wen et al., 2022; Yan et al., 2022). However, this study will aim to test the effectiveness of Tai Chi in treating insomnia in patients with CNLBP.

This trial will have several strengths that increase its validity and reliability. First, the randomized controlled trial design of the study allows for a fair comparison of the effects of Tai Chi on insomnia in patients with CNLBP. Second, the study takes into account multiple factors that affect sleep quality, such as physical, psychological, and social factors. Additionally, the protocol adheres to CONSORT and SPIRIT guidelines.

However, this study will also face some limitations. First, the high expectations of the participants in the Tai Chi group may influence the results. Second, there is a risk of high dropout rates during the waiting period. Finally, the research process may be affected by COVID-19.

The results of this trial may help improve sleep quality and reduce pain in people with CNLBP. Furthermore, they could advocate for the promotion of Tai Chi training as a therapeutic option to treat insomnia in elderly individuals with CNLBP. Tai Chi may be considered a viable therapeutic option for CNLBP patients with insomnia.

## Ethics statement

This study was approved by the Ethics Committee of the Hospital of Chengdu University of Traditional Chinese Medicine (2022KL-038-02). The patients/participants provided their written informed consent to participate in this study.

## Author contributions

FZ and BZ wrote the draft manuscript. FZ designed the trial and all data will be double-checked. XW carried out the statistical analysis plan. BH performed the data collection. CH will record the data using the REDCap. All authors read and approved the final version of the manuscript.
